# Sites of Glucose Transporter-4 Vesicle Fusion with the Plasma Membrane Correlate Spatially with Microtubules

**DOI:** 10.1371/journal.pone.0043662

**Published:** 2012-08-20

**Authors:** Jennine M. Dawicki-McKenna, Yale E. Goldman, E. Michael Ostap

**Affiliations:** 1 Pennsylvania Muscle Institute, Perelman School of Medicine at the University of Pennsylvania, Philadelphia, Pennsylvania, United States of America; 2 Department of Physiology, Perelman School of Medicine at the University of Pennsylvania, Philadelphia, Pennsylvania, United States of America; NHLBI, NIH, United States of America

## Abstract

In adipocytes, vesicles containing glucose transporter-4 (GLUT4) redistribute from intracellular stores to the cell periphery in response to insulin stimulation. Vesicles then fuse with the plasma membrane, facilitating glucose transport into the cell. To gain insight into the details of microtubule involvement, we examined the spatial organization and dynamics of microtubules in relation to GLUT4 vesicle trafficking in living 3T3-L1 adipocytes using total internal reflection fluorescence (TIRF) microscopy. Insulin stimulated an increase in microtubule density and curvature within the TIRF-illuminated region of the cell. The high degree of curvature and abrupt displacements of microtubules indicate that substantial forces act on microtubules. The time course of the microtubule density increase precedes that of the increase in intensity of fluorescently-tagged GLUT4 in this same region of the cell. In addition, portions of the microtubules are highly curved and are pulled closer to the cell cortex, as confirmed by Parallax microscopy. Microtubule disruption delayed and modestly reduced GLUT4 accumulation at the plasma membrane. Quantitative analysis revealed that fusions of GLUT4-containing vesicles with the plasma membrane, detected using insulin-regulated aminopeptidase with a pH-sensitive GFP tag (pHluorin), preferentially occur near microtubules. Interestingly, long-distance vesicle movement along microtubules visible at the cell surface prior to fusion does not appear to account for this proximity. We conclude that microtubules may be important in providing spatial information for GLUT4 vesicle fusion.

## Introduction

GLUT4 is a facilitative glucose transporter important in the uptake of glucose into fat and muscle tissue in response to insulin [1], [2]. Because of the importance of GLUT4 in maintaining blood glucose homeostasis, its intracellular localization and plasma membrane insertion are highly regulated. Under basal conditions, the majority of GLUT4 is sequestered within a specialized, insulin-sensitive storage compartment in the form of vesicles or tubulo-vesicular structures [3], [4]. The intracellular pool of GLUT4 is dynamic, and the basal distribution reflects fast endocytosis from the plasma membrane [5]–[7] and slow exocytosis of GLUT4-containing vesicles [5]–[8]. This results in a low level of plasma membrane-inserted GLUT4 in the basal state. Insulin binding to the insulin receptor begins a series of signaling events, which culminate in a substantial increase in the rate of exocytosis [5]–[8].

The insertion of GLUT4 into the plasma-membrane occurs through a multi-step process involving redistribution of vesicles to the cell periphery, where the vesicles tether, dock, and fuse (reviewed in [9]–[13]). There is an increasing appreciation that both the actin and microtubule cytoskeletons participate in this process (reviewed in [13], [14]). Microtubules are thought to be involved in a pre-fusion step, and disruption of the microtubule cytoskeleton decreases the fraction of surface-localized GLUT4 by ∼40% [8], [15]. Actin [16], myosin-I [17]–[19], myosin-V [20], [21] and myosin-II [22], [23] have been implicated in the final steps of GLUT4 trafficking occurring at the plasma membrane.

Tracking and mobility analysis examining the movements of individual GLUT4 vesicles in live cells has provided further evidence for a role of microtubules in GLUT4 trafficking. However, in many cases, the studies do not directly distinguish individual GLUT4 vesicles that ultimately fuse with the plasma membrane in response to insulin from those that remain intracellular. GLUT4-containing vesicles move laterally distances greater than 1 µm [15], [24]–[27], the movements co-localize with microtubules [24], [25], and long-distance movement requires intact microtubules [15], [24], [26]. Analyses to assess the mobility of GLUT4 vesicles, for example fluorescence recovery after photobleaching, revealed that vesicles are mobile in the basal state and that microtubule disruption reduces basal mobility [26], [28].

Several microtubule motors have been identified that could mediate GLUT4 vesicle movement along microtubules. Rab5, Rab4, and the scaffolding protein Daxx, present on GLUT4 vesicles, have been identified as insulin-sensitive interacting partners for dynein, a kinesin II (KIF3), and kinesin I (KIF5B), respectively [29]–[31]. Disruption of the plus-end directed motor KIF3 through antibody injection [30], or conventional kinesin, through over-expression of a dominant-negative mutant of conventional kinesin light chain [24], decreases GLUT4 translocation in response to insulin.

While it is clear that microtubules are involved in GLUT4 vesicle motility, whether microtubule-based GLUT4 vesicle movement is required for the insulin-induced GLUT4 redistribution to the plasma membrane is not known. Insulin may regulate GLUT4 vesicle engagement or movement along microtubules as suggested by work in 3T3-L1 adipocytes, in which the fraction of mobile vesicles and vesicle speed increased upon insulin stimulation [27]. However, others observed halting of vesicle trafficking along microtubules in response to insulin in primary rat adipocytes [25]. An absolute requirement for an insulin-stimulated increase in GLUT4 mobilization along microtubules has been challenged by experiments showing that expression of constitutively active Akt, a serine/threonine protein kinase, is sufficient even in the absence of an intact microtubule cytoskeleton to redistribute GLUT4 in response to insulin [28]. In light of these findings, we hypothesize that long-distance movement of GLUT4 vesicles is not the main role for microtubules in GLUT4 trafficking.

To gain insight into the molecular role of microtubules in GLUT4 exocytosis, we examined the arrangement and dynamics of microtubules in 3T3-L1 adipocytes using live-cell total internal reflection fluorescence (TIRF) imaging. We show that insulin increases the density and dynamics of microtubules at the cell surface. Disruption of microtubules with 10 µM nocodazole decreases the rate and extent of accumulation of GLUT4 at the plasma membrane, suggesting impairment in GLUT4 translocation in the absence of microtubules. Interestingly, fusions of GLUT4-containing vesicles with the plasma membrane preferentially occur in proximity to microtubules, and long-distance vesicle movement along microtubules visible at the cell surface prior to fusion does not appear to account for this proximity. We hypothesize that microtubules may be important in specifying sites of GLUT4 vesicle fusion with the plasma membrane. For example, microtubules could be serving as scaffolds for the organization of signaling or fusion machinery.

## Results

### 3T3-L1 Adipocytes Redistribute GLUT4 in Response to Insulin

**Figure 1:**
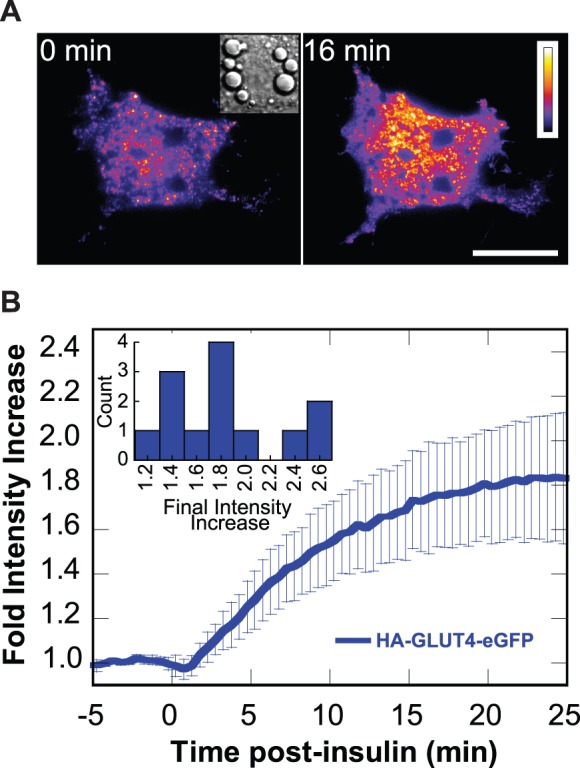
Insulin stimulation increases the intensity of HA-GLUT4-eGFP within the TIRF illumination zone. Adipocytes transfected with HA-GLUT4-eGFP were serum-starved prior to stimulation with 100 nM insulin at t = 0 min. Images were acquired using TIRF microscopy at 1 frame per 10 s. (A) Pseudocolor of HA-GLUT4-eGFP intensity before and after insulin stimulation. Elapsed time from insulin addition is indicated. Scale bar is 20 µm. The calibration bar at left indicates HA-GLUT4-GFP intensity with black and white the least and greatest intensities, respectively. See Video S1. (Inset) Brightfield image. Box is 27 µm×27 µm. (B) Time course of the increase in fluorescence intensity in response to insulin. Fold intensity increase relative to the average intensity prior to insulin addition was calculated for each cell. Plotted is the mean ±95% confidence interval (n = 13 cells). (Inset) Histogram of the fold intensity change at plateau.

TIRF microscopy revealed a striking insulin-dependent movement of fluorescently-tagged GLUT4 (HA-GLUT4-eGFP) from internal compartments to the coverslip-attached surface of adipocytes (Figure 1A, Video S1). The average fluorescence intensity from cells that exhibited a >10% enhancement (Figure 1B inset) reached a plateau with a half-time (*t*_1/2_) of 6.3 min (Table 1), which is similar to previously observed time courses [32]–[34]. Time courses are similar regardless of the final fold intensity increase (Figure S1C). The 1.8-fold average increase in fluorescence intensity (Figure 1B inset, Table 1) is consistent with previous reports of average fluorescence increase of between ∼1.5 to 3-fold in response to insulin in studies monitoring GLUT4-GFP translocation using TIRF microscopy [32]–[35]. Control experiments, exchanging media without insulin, confirm that the fluorescence change is the result of the addition of insulin (Figure S1A). In addition, since intensity increase is measured only in the initial cell footprint, the observed intensity increase is not a reflection of the attached surface area increase that occurs in some cells in response to insulin.

**Table 1:** Time course parameters.

CONDITION	Max fold±95%CI^a^	*t*_1/2_(min)	*t*_lag_(min)	n^b^
HA-GLUT4-eGFP, 0 µM Noc	1.8±0.3	6.3	0.8	13
HA-GLUT4-eGFP, 10 µM Noc	1.6±0.3	9.8	3.5	7
IRAP-pHluorin, 0 µM Noc	2.8±1.1	7.0	1.4	8
IRAP-pHluorin, 10 µM Noc	2.1±0.5	8.5	1.7	12
Microtubule density	1.4±0.1	2.5	0.7	18

**Figure 2:**
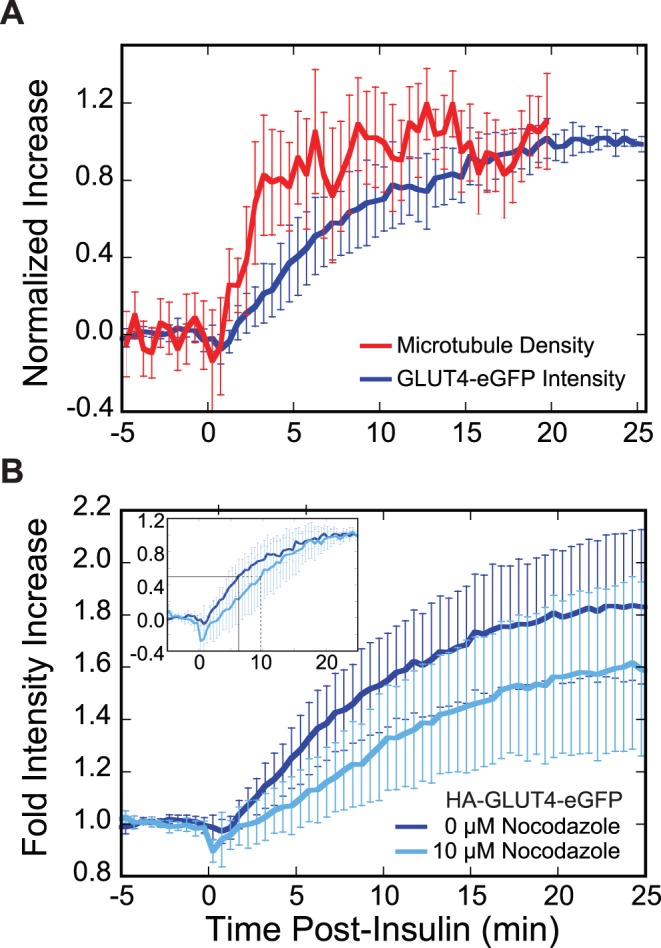
Time courses of HA-GLUT4-eGFP intensity and microtubule density increase. (A) Insulin increases microtubule density in the region of the cell illuminated by TIRF microscopy. Adipocytes transfected with constructs either to visualize the GLUT4 or microtubule time course were serum-starved prior to stimulation with 100 nM insulin at t = 0 min. Images were acquired using TIRF microscopy at 1 frame per 10 s. Intensity or density increase for each cell was normalized from 0 (average intensity or density prior to insulin addition) to 1 (intensity at last 1 minute or density at last 8 minutes of time course). Plotted is the mean ±95% confidence interval for each point (HA-GLUT4-eGFP, n = 13 cells; mCherry-tubulin, n = 18 cells). (B) Nocodazole pre-treatment decreases the fold change in GLUT4 intensity in response to insulin. Adipocytes expressing HA-GLUT4-eGFP were serum-starved and pre-treated with 0 µM (dark blue, n = 13 cells) or 10 µM (light blue, n = 7 cells) nocodazole for a minimum of 20 min prior to stimulation with 100 nM insulin at t = 0 min. Images were acquired using TIRF microscopy at 1 frame per 10 s. Plotted is the time course of the mean fold intensity increase ±95% confidence interval. There is a significant difference (two-tailed t-test; p-value <0.05) between the two time courses at the half-time for HA-GLUT4-eGFP intensity increase (t = 6.3 min, 0 µM Noc). Due to heterogeneity in the magnitude of the insulin response (Figure 1B inset), a two-tailed t-test performed at t = 25 min does not yield a significant p-value (p-value >0.05). However, since there is a temporal relationship between time points, a new p-value, P, can be calculated by taking this relationship into consideration (see Methods). Comparing the last several minutes of the plateaus gives a significant difference (P<0.05) between the 0 µM Noc and 10 µM Noc time courses. (Inset) Time course replotted to show the intensity increase normalized from 0 (average intensity prior to insulin addition) to 1 (intensity at last minute of time course). Plotted is the mean ±95% confidence interval. Half-times are plotted (0 µM Noc, *t*_1/2_ = 6.3 min, solid black line; 10 µM Noc, *t*_1/2_ = 9.8 min, dashed black line).

Time course parameters are calculated from Figures 1–2, and S5.

a Max fold intensity or density increase relative to baseline. CI, confidence interval.

b Number of cells.

### Microtubule Density Increases in Response to Insulin Stimulation in 3T3-L1 Adipocytes

**Figure 3:**
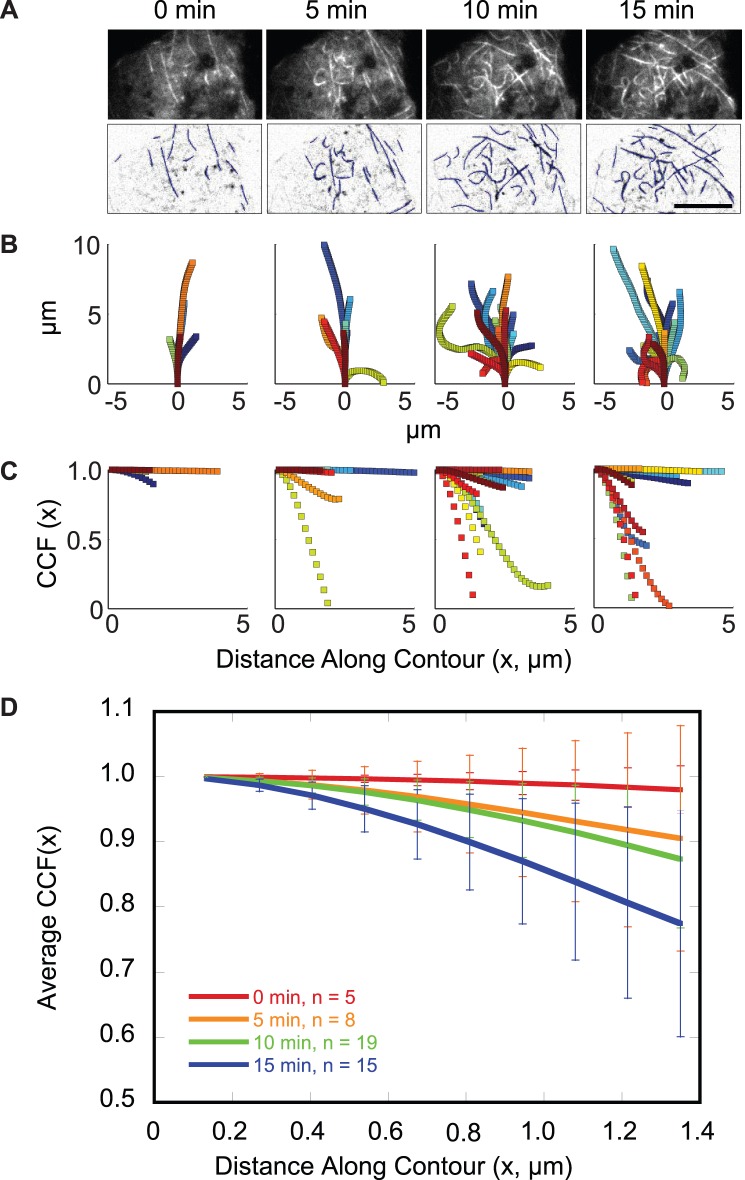
TIRF microscopy reveals a population of highly curved microtubules at the surface of 3T3-L1 adipocytes. Adipocyte transfected with mCherry-tubulin was serum-starved prior to stimulation with 100 nM insulin at t = 0 min. Images were acquired using TIRF microscopy at 1 frame per 10 s. Elapsed time from insulin addition is indicated. See Video S2. (A) (Upper) Unprocessed and (Lower) background-subtracted, inverted contrast images of an adipocyte at the indicated times following insulin stimulation. Microtubule contours are overlaid in blue. Scale bar is 10 µm. (B) Contours for microtubules at least 3 µm in length were replotted to share the same origin (0 min, 5 contours; 5 min, 8 contours; 10 min, 19 contours; 15 min, 15 contours). (C) Cosine correlation function for microtubule contours shown in (B), see supplement for details (Materials S1: Cosine correlation function, Figure S3). (D) The average CCF up to the minimum contour length for microtubule contours shown in (C) are plotted at 0 (red), 5 (orange), 10 (green), and 15 (blue) minutes post-insulin. Error bars represent the 95% confidence interval.

The density of microtubules within the TIRF illumination zone increased substantially upon insulin stimulation, as detected by the localization of mCherry-tubulin (Figures 2A and 3A, Table 1, Videos S2 and S3), GFP-tubulin (Figure 2A, Table 1), or 3xGFP-EMTB, the microtubule-binding domain of ensconsin (Figure 2A, Table 1). This intensity increase occurred with a half-time (*t*_1/2_ = 2.5 min) that was 2.5-fold faster than the HA-GLUT4-eGFP intensity increase (*t*_1/2_ = 6.3 min; Figure 2A, Table 1). The insulin-stimulated increase of microtubule density was also observed in cells co-expressing HA-GLUT4-eGFP and mCherry-tubulin (Figure S1B).

Treatment of cells with 10 µM nocodazole for 20 min, which results in large-scale depolymerization of the microtubule cytoskeleton (data not shown), reduced the insulin-stimulated increase in HA-GLUT4-eGFP intensity from 1.8-fold in the absence of nocodazole to 1.6-fold at 10 µM nocodazole (Figure 2B, Table 1). Nocodazole treatment also introduced a time-lag (*t*_lag_ = 3.5 min), defined as the intercept with the baseline of a tangent line drawn through the inflection point, and slowed the kinetics of the fluorescence increase (*t*_1/2_ = 9.8 min) in the TIRF-zone (Figure 2B inset).

### Insulin-stimulation Induces Microtubule Curvature

Strikingly, a population of highly curved microtubules is detected in the TIRF-illumination zone of adipocytes. Curvature is detected by immunofluorescence of endogenous microtubules as well as by live-cell imaging of adipocytes transfected with GFP-tubulin, mCherry-tubulin, or 3xGFP-EMTB (Figures 3A and S2A, Videos S2 and S3). When plotted so that the coordinates of continuous microtubule segments ≥3 µm in length share the same origin and beginning orientation, the curvature appears to increase upon insulin stimulation (Figure 3B). We quantified curvature by calculating the cosine correlation function (CCF) along the contour of the microtubule (Figures 3C and S3B, see supplement for details). The CCF of more highly curved microtubules decays toward 0 more quickly than straighter microtubules. We plotted the average CCF for microtubule segments at least 3 µm in length as a function of time, and found a trend towards increased curvature with time after insulin stimulation (Figure 3D). Because of difficulty in quantifying the data, we cannot say with statistical certainty that curvature increases after insulin stimulation (two-tailed t-test; p = 0.17). However, it is clear that the number of curved microtubules at the membrane increases.

**Figure 4:**
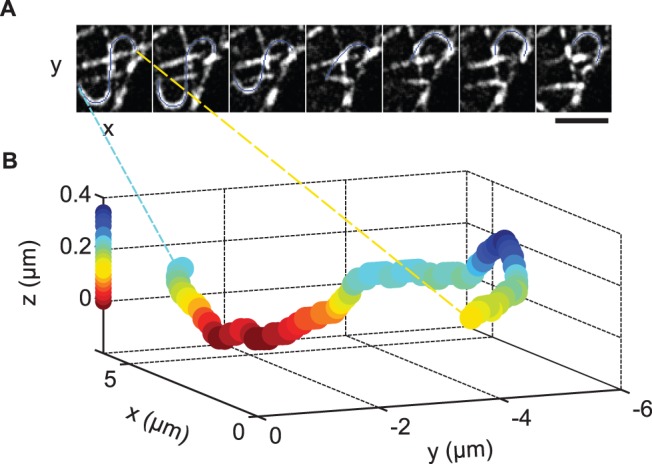
Curved regions of microtubules are actively displaced. Adipocyte was transfected with 3×GFP-EMTB in order to visualize microtubules. Images were acquired using TIRF microscopy. (A) Force-induced displacement of a microtubule. Images from a time course (Videos S7) have been background-subtracted. Frames are displayed at 30 s intervals. Microtubule contour is overlaid in blue. Scale bar is 3 µm. (B) 3-Dimensional imaging of curved microtubule using Parallax and TIRF microscopies. The microtubule displayed in (A, first frame) is plotted as a series of (x,y,z) coordinates. A pair of 2-dimensional TIRF images (A: 1 of pair) was used to calculate relative z-depth. Warmer colors are closer to the coverslip, as indicated by the calibration bar along the z-axis. For an animation of the 3-dimensional time course see Video S8.

Many curved microtubules appeared to be actively and abruptly displaced by forces that translocated them along the plane of the membrane or pulled them closer to or further from the cell surface (Figure 4, Videos S4,S5,S6,S7,S8). Several types of motion were observed. For example, microtubule loops often formed when an internal region of a microtubule moved parallel to the x–y plane. In some instances, the locations where the microtubule transitioned to visibility in TIRF appeared fixed, as if the microtubule was being threaded through these locations near the cell surface (Videos S4). Microtubules also appeared to move relative to or slide along a second, more stationary microtubule or microtubule segment (Video S5). In other cases, straight segments of microtubules appeared to glide in a plane parallel to the coverslip (Video S6). Three-dimensional time-lapse imaging using Parallax microscopy [36] revealed abrupt, short duration z-displacements of microtubule regions as they moved toward and away from the plasma membrane (Figure 4, Videos S7 and S8).

To investigate whether microtubule polymerization could account for the observed microtubule bending, we treated adipocytes with a low dose of nocodazole (2 µM), and examined microtubule dynamics using TIRF microscopy. Under these conditions, tubulin heterodimers are sequestered, resulting in microtubule depolymerization (Video S9). However, substantial curvature and displacement dynamics remain (Figure S2B, Video S9), suggesting that forces generated from polymerization are not driving curvature.

We next tested whether microtubule-based motor activity or actin-dependent motility is responsible for the observed microtubule dynamics. Over-expression of a dominant-negative construct of p150^Glued^ (GFP-CC1) to disrupt dynein-dynactin function [37], [38] did not abolish the microtubule curvature or the forces displacing the microtubules (Figure S2C), nor did treatment with either 2 µM cytochalasin D (cytoD) or 20 µM latrunculin B (latB) to disrupt the actin cytoskeleton (Figure S2D). These results suggest the involvement of a kinesin motor.

Curvature is not correlated with microtubule acetylation (Figure S4A), in contrast to what has been found in other cell types [39]. Microtubules in adipocytes appeared to have greater levels of acetylation than microtubules in incompletely differentiated 3T3-L1 cells found on the same coverslip (Figure S4B). As reported previously, more centrally-localized microtubules had higher acetylation levels than peripheral microtubules (Figure S4A–B) [39].

### Fusion Events Preferentially Occur Near Microtubules

**Figure 5:**
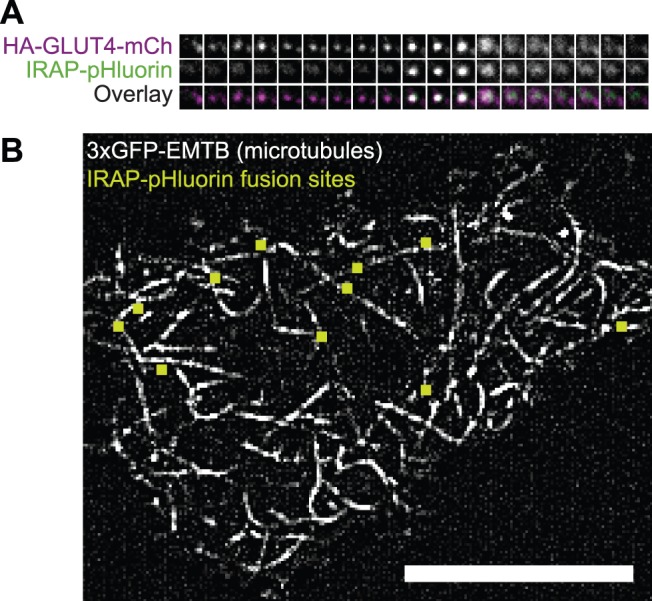
Sites of IRAP-pHluorin vesicle fusion occur in proximity to microtubules at the cell surface. Adipocytes co-transfected with the indicated constructs were serum-starved prior to stimulation with 100 nM insulin. Images were acquired using TIRF microscopy at an acquisition rate of 20 frames per 1 s. (A) Fusion of a single GLUT4 vesicle with the plasma membrane. Vertical panels represent consecutive frames of the recording. Each horizontal panel displays a single channel of a Dual-View image: (Top) HA-GLUT4-mCherry, (Middle) IRAP-pHluorin, (Bottom) Overlay. Each box is 1.89 µm×1.89 µm. (B) Fusions occur in proximity to microtubules visible in TIRF microscopy. Displayed is a 50 frame (2.5 s) maximum intensity projection image of an adipocyte co-expressing mCherry-IRAP-pHluorin and 3×GFP-EMTB. The image has been background-subtracted to better visualize microtubules. Sites of vesicle fusion during the 2.5 s are overlaid (green squares). Scale bar is 10 µm. See Video S10.

Both HA-GLUT4-eGFP-containing vesicles in proximity to the cell surface, and HA-GLUT4-eGFP inserted in the plasma membrane, contribute to the observed TIRF fluorescence intensity. To better detect fusion of vesicles with the plasma membrane, we used a construct (IRAP-pHluorin) with the pH-sensitive eGFP variant (pHluorin) [40] positioned on the lumenal side of amino acids 1–393 of insulin-regulated aminopeptidase (IRAP) [41]. This construct uses IRAP as a surrogate for GLUT4 since fluorophores on a lumenal domain of GLUT4 typically disrupt GLUT4 trafficking. IRAP is a transmembrane protein enriched in insulin-responsive vesicles that co-localizes and co-fractionates with GLUT4 [42]–[46] and traffics in a manner indistinguishable from GLUT4 [47]. The lumenal pH of GLUT4 vesicles, and, therefore, pHluorin fluorescence emission, is low. Upon fusion of GLUT4 vesicles with the plasma membrane, pHluorin is exposed to the neutral pH of the imaging medium, and the fluorescence emission increases. To demonstrate the improved fusion detection, we co-expressed HA-GLUT4-mCherry and IRAP-pHluorin and simultaneously excited the fluorophores. The time course of a single vesicle fusion event is shown (Figure 5A), beginning with the initial approach of the vesicle to the cell surface and ending with dispersal within the plasma membrane.

We confirmed that IRAP-pHluorin TIRF intensity increases in response to insulin (Figure S5A, Table 1). IRAP-pHluorin intensity increases within the first few minutes of insulin addition with a *t*_1/2_ = 7 min. The time to plateau is slightly longer than measured for HA-GLUT4-eGFP, which may reflect the selective detection of membrane fusion events, rather than proximity to the plasma membrane. Interestingly, 10 µM nocodazole treatment to disrupt microtubules did not markedly slow the time course of the IRAP-pHluorin fluorescence increase (Figure S5B, Table 1).

**Figure 6:**
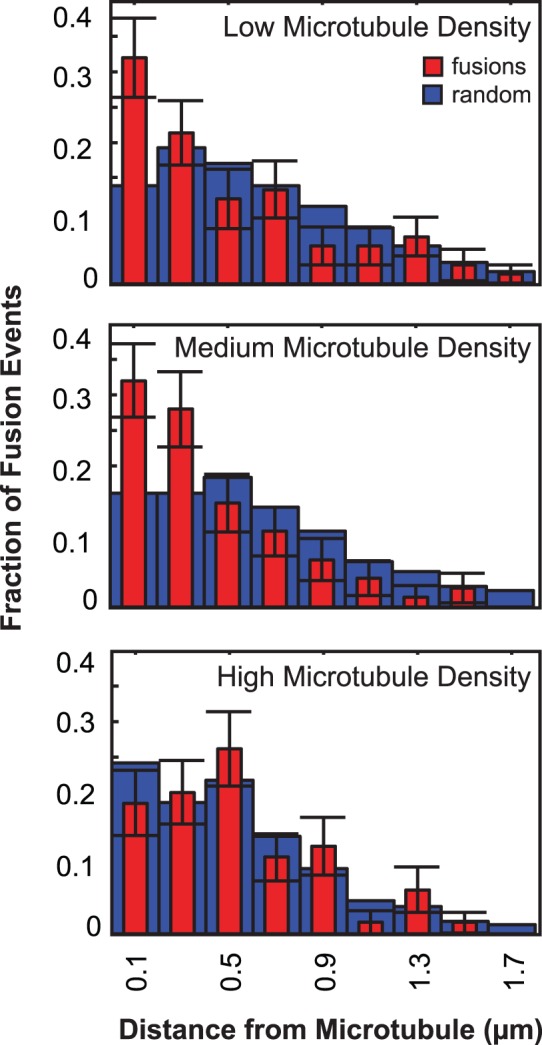
Sites of IRAP-pHluorin fusion are spatially correlated with microtubules present in the TIRF illumination zone. Adipocytes co-transfected with IRAP-pHluorin and mCherry-tubulin were serum-starved prior to stimulation with 100 nM insulin. Histogram of distance of fusion events from a microtubule obtained using TIRF microscopy and a Dual-view insert at an acquisition rate of 20 frames per 1 s (red, fusions; blue, random locations). Center of bin (width = 0.2 µm) is indicated. Error bars represent the standard deviation resulting from bootstrapping (n = 100 repetitions) the fusion data.

Sites of insulin-stimulated vesicle fusion appear to correlate spatially with microtubules in the TIRF-illumination zone. Simultaneously imaged mCherry-IRAP-pHluorin and 3xGFP-EMTB in adipocytes show that that many fusion events occur in proximity to microtubules (Figure 5B, Video S10). To obtain a quantitative measure of proximity, we correlated the position of microtubules with fusion sites by simultaneously imaging mCherry-tubulin and IRAP-pHluorin. While there is no observable difference in GLUT4 vesicle behavior in cells of different microtubule densities, microtubule density will influence the random distribution because the probability that a fusion will occur near a microtubule depends on the microtubule density. Therefore, proximity distances were determined at several cellular microtubule densities. A cumulative distribution of the distance of fusion events in cells with low microtubule density shows that ∼40% of fusions occur ≤200 nm (approximately the resolution of our measurement) from a microtubule (Figure 6). Similarly, at an intermediate microtubule density, the probability of a fusion occurring adjacent to a microtubule is substantially greater than random chance. At high densities, even random sites occur near microtubules (Figure 6). We have not determined whether a spatial correlation exists between microtubules and fusions of other types of vesicles.

### Long-distance Movement of mCherry-IRAP-pHluorin Prior to Vesicle Fusion is Only Rarely Detected

**Figure 7:**
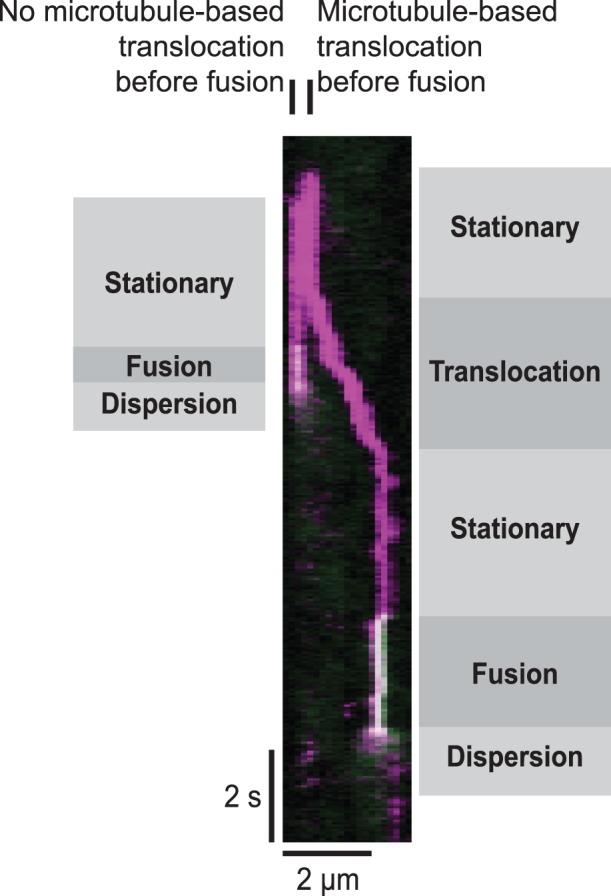
Movement of vesicles long distances prior to fusion is observed infrequently. Adipocyte transfected with mCherry-IRAP-pHluorin was serum-starved prior to stimulation with 100 nM insulin. Images were acquired using TIRF microscopy at an acquisition rate of 20 frames per 1 s, and a kymograph was generated. mCherry (magenta) allows visualization of the vesicle prior to fusion with the plasma membrane, at which time the pHluorin (green) intensity increases. The overlay of magenta and green appears white. The vesicle on the right represents an example of a rare vesicle fusion event that is preceded by a long-distance movement. On the left, a second vesicle approaches the surface in the same vicinity as the first but fuses without detected lateral movement.

Given that exocytic events preferentially take place in proximity to microtubules visible at the cell surface, we asked whether vesicle movements prior to fusion were consistent with microtubule-based transport. In particular, we asked whether vesicles moved linear distances at least ∼1 µm prior to fusion. Cells were transfected with mCherry-IRAP-pHluorin, a construct which allows us to both observe vesicles prior to fusion (mCherry) and detect fusions efficiently (pHluorin). We observed that long-distance movements detectable using TIRF microscopy (Figure 7) rarely precede vesicle fusion in untreated cells (Table 2) and could not be detected in nocodazole-treated cells (data not shown). Most often, vesicles appeared to approach the cell surface vertically without appreciable directed lateral movement, although some vesicles appeared to change velocity and direction repeatedly and rapidly prior to immobilization and fusion. These movements occurred in both untreated and nocodazole-treated cells (data not shown). We included in our analysis only those vesicles that we observed entering the TIRF illumination zone, eliminating the possibility that our analysis includes vesicles whose long-distance movement we failed to detect due to the timing of the acquisition.

**Table 2:** Vesicle movement prior to fusion with the plasma membrane.

TOTAL	Unknown^a^	Fusions preceded by ∼1 µm linear displacement	Fusions in which linear displacement was undetected^b^
221	47	4	170

Vesicle movements prior to fusion with the plasma membrane were determined in 3T3-L1 adipocytes (n = 4 cells) transfected with mCherry-IRAP-pHluorin and stimulated with 100 nM insulin.

a Vesicle movements were scored as unknown when high local vesicle density or low mCherry fluorescence intensity made determination of movement difficult.

b Linear displacement of ∼1 µm or more was undetected prior to fusion of vesicle.

## Discussion

### Microtubule Curvature and Surface Density Increase May be Mechanistically Linked

The increases in microtubule density and bending dynamics in the TIRF illumination zone are striking down-stream effects of insulin signaling (Figures 2A and S1B). While it has been shown previously that the total amount of polymerized tubulin increases upon insulin stimulation [48], the dramatic increase in concentration (Figure 2A) and bending of the filaments we observed at the cell membrane (Figure 3) was unexpected since there remains a significant amount of cytoplasm outside of the TIRF-illuminated region of the cell despite the abundant lipid droplets in 3T3-L1 adipocytes. The correlated increase in density and bending (Figure 3D) may be caused by related mechanisms, as the microtubules appear to be forcibly pulled toward the cell surface (Figure 4, Videos S4,S5,S6,S7,S8, see below).

Cells treated with a nocodazole concentration that inhibits microtubule polymerization, but does not result in complete depolymerization, showed persistent microtubule curvature and pulling, indicating that the dynamics are not the result of polymerization-induced buckling (Figure S2B, Video S9). Likewise, disruption of the actin cytoskeleton (Figure S2D) or dynein-dynactin function (Figure S2C) did not appear to diminish the microtubule curvature or the microtubule displacements. Despite extensive disruption of the actin cytoskeleton with latrunculin B, it is possible that a population of actin filaments remained intact and were capable of supporting actomyosin-based contractility, which contributed to the curvature. However, considering that microtubule sliding occurs relative to other microtubules (Video S5) and that gliding of microtubules can be observed along the surface of adipocytes (Video S6), we believe a microtubule-based motor to be involved. Kinesin motors may be responsible for microtubule bending. One candidate motor that should be considered is kinesin-1 heavy chain, which is involved in microtubule-microtubule sliding in Xenopus and Ptk2 cells [49].

### Microtubules are not Required but Contribute to the Dynamics of the GLUT4 Insulin Response

The increase in microtubule density (*t*_1/2_ = 2.5 min) at the cell membrane preceded the increase in GLUT4 intensity (*t*_1/2_ = 6.3 min; Figure 2A), so it is interesting to ask if microtubules have a role in the transport of GLUT4 to the cell surface or to the distribution of fusion sites. Nocodazole disruption of microtubules delayed the half-time for insulin-stimulated accumulation of GLUT4 at the cell surface from a *t_1/2_* = 6.3 min in the absence of nocodazole to *t_1/2_* = 9.8 min at 10 µM nocodazole (Table 1) and introduced a time-lag in the intensity increase (Figure 2B inset, Table 1). Nocodazole also modestly decreased the magnitude of GLUT4 accumulation at the membrane (Figure 2B, Table 1), consistent with several previous studies [8], [48], [50]–[52]. GLUT4 vesicle trafficking is highly regulated, so it is unlikely that microtubule stabilization is sufficient by itself to drive GLUT4 redistribution to the plasma membrane in the absence of insulin signaling. In support of this supposition, microtubule stabilization with taxol does not stimulate an increase in the basal level of GLUT4 at the cell surface [53].

There is controversy in the literature over the role of microtubules in GLUT4 vesicle trafficking. Some have argued that transport of GLUT4 vesicles along microtubules is required for GLUT4 redistribution in response to insulin [26]. In support of this claim, single vesicle analysis indicates a decreased vesicle density underneath the plasma membrane when microtubules are disrupted [26]. In addition, some studies report a dramatic decrease in insulin-stimulated GLUT4 redistribution to the cell surface following microtubule disruption [26], [54]. However, others argue that while microtubules play a role in basal mobility of GLUT4 vesicles [26], [28], long-distance movement of GLUT4 vesicles along microtubules is not required for redistribution to the cell surface [28]. In contrast to the observations above, several studies show only a modest effect of microtubule disruption on GLUT4 vesicle trafficking in response to insulin [8], [15], [48], [52]). Furthermore, expression of constitutively active Akt induces GLUT4 to redistribute to the plasma membrane even when microtubules are disrupted [28].

Our results are consistent with studies showing only a mild impairment of GLUT4 translocation upon microtubule disruption (Figure 2B, Table 1). Microtubules may be playing a modest role in the effectiveness of mobilizing GLUT4 vesicles to the surface. They are also important for appropriate sequestering of GLUT4 in the basal state [52]. However, there appears to be no absolute requirement for microtubules in increasing the plasma membrane insertion of GLUT4, as indicated by the increase in IRAP-pHluorin intensity in response to insulin in nocodazole-treated cells (Figure S5, Table 1). Therefore, microtubules do not appear to be required for transport of GLUT4 vesicles to the plasma membrane for fusion on the time scale of these experiments.

### Microtubules may be Specifying Sites of GLUT4 Vesicle Fusion with the Plasma Membrane

It is clear that fusions of GLUT4 vesicles with the plasma membrane detected using IRAP-pHluorin occur more closely to microtubules than would be expected for a random distribution (Figure 6). Long-distance GLUT4 vesicle movements can readily be observed at the surface of 3T3-L1 adipocytes [24], [26], [27]. However, we observed that only a small fraction of long-distance movements at the surface resulted in vesicle fusion during our observation time (data not shown). In addition, using TIRF microscopy, we rarely detected directed transport of GLUT4 vesicles that persisted for more than 1 µm along the filaments prior to fusion (Figure 7, Table 2). Long-distance movement on microtubules at the cell surface, therefore, does not appear to account for vesicle localization just prior to fusion in 3T3-L1 adipocytes. The situation may be different in primary rat adipocytes where insulin halts GLUT4 vesicle trafficking along microtubules and promotes vesicle fusion with the plasma membrane [25].

As microtubules at the membrane appear to be more important for specifying fusion site locations than for transporting GLUT4 vesicles, they could be involved in the delivery, localization, or scaffolding of signaling components or fusion machinery. In support of this hypothesis, results from Eyster et al. suggesting that microtubule disruption inhibits Akt activation imply a role for microtubules in organizing the insulin signaling complex [28]. Microtubules have also been proposed to regulate phosphatidylinositol 3-kinase activity during phagocytosis in macrophages [55], a process that shares with insulin-induced GLUT4 trafficking a requirement for Akt membrane recruitment and activation.

That microtubules could be playing a role in specifying or assembling fusion sites is an interesting possibility, and more work needs to be done to investigate this hypothesis. One potential approach is to examine whether a spatial correlation exists between microtubules and proteins involved in the initial interaction of GLUT4 vesicles with the plasma membrane. For instance, in primary adipocytes, Exo70, a component of the exocyst complex, has been proposed to tether GLUT4 vesicles at the plasma membrane prior to fusion, preventing GLUT4 vesicle translocation along surface-localized microtubules [56].

## Materials and Methods

### Reagents and Cloning

Tissue culture reagents were from Gibco, except fetal bovine serum (FBS) and recombinant, human insulin, which were from Sigma. Nocodazole, cytochalasin D, and latrunculin B were obtained from both A.G. Scientific and Sigma. Radioimmuno assay-grade bovine serum albumin (RIA-BSA) (Calbiochem) was used for serum starvation.

mCherry-IRAP-pHluorin (pJDM19) was constructed by inserting mCherry (Dr. R. Tsien, [57]) prior to the N-terminal 393-bp fragment of IRAP in the IRAP-pHluorin construct (Dr. D. James, [41]) using Nhe I and Bgl II restriction sites. HA-GLUT4-mCherry (pJDM12) was constructed by substituting mCherry for eGFP in the HA-GLUT4-eGFP construct (Dr. S. Cushman, [58]) using KpnI and XbaI restriction sites. See supplement for PCR primers used to generate restriction sites.

### Cell Culture, Transfection, and Live-cell Imaging

Mouse 3T3-L1fibroblasts [59] (source: Dr. M. Birnbaum, University of Pennsylvania) were cultured in growth medium (high glucose (4.5 g/L) DMEM +10% FBS supplemented with antibiotics) in an incubator at 37°C, 5% CO_2_. Fibroblasts were differentiated into adipocytes in differentiation medium (DMEM, 10% FBS, 0.5 mM isobutylmethylxanthine, 400 ng/mL dexamethasone, 400 nM insulin, and 10 µg/mL troglitazone) essentially as described [47]. Adipocytes were used at 6–10 days post-differentiation for live cell imaging or 7–14 days for fixed cell imaging. Morphologically, we considered cells with prominent lipid droplets to be adipocytes (Fig. 1A inset). These cells were generally more rounded than cells lacking lipid droplets, although adipocytes became somewhat less rounded following replating of the cells for imaging.

For transient transfection, 3T3-L1 adipocytes were trypsinized (0.25% trypsin-EDTA), washed twice, and resuspended in phosphate-buffered saline without Ca^2+^ or Mg^2+^. DNA and cells were added to a 0.4 cm electroporation cuvette in a final volume of ∼700 µL. Cells were electroporated at 200 V, 950 µF and re-plated onto acid-washed coverslips. The amount of DNA used per transfection varied by construct: HA-GLUT4-eGFP, 80 µg; IRAP-pHluorin, 20–25 µg; eGFP-human-α-tubulin (Clontech), 40 µg; mCherry-human-α-tubulin (Dr. R. Tsien, [57]), 40 µg; 3×GFP-EMTB (ensconsin microtubule-binding domain) (Dr. C. Bulinski, [60]), 5 µg; mCherry-IRAP-pHluorin, 10 µg; GFP-CC1 p150^Glued^ (Dr. E. Holzbaur, [38]), 60 µg. Cells were imaged 24–48 hours following transfection.

Prior to live-cell imaging, adipocytes were serum-starved for 2 - ∼12 hours in either Krebs Ringer Phosphate buffer (136 mM NaCl, 4.7 mM KCl, 10 mM NaPO_4_, pH 7.4, 0.49 mM MgCl_2_, and 0.9 mM CaCl_2_) +0.2% RIA-BSA or high glucose DMEM +0.5% RIA-BSA. Where indicated, cells were pre-treated with 10 µM nocodazole for a minimum of 20 minutes. Coverslips were then transferred to an enclosed Bioptechs chamber (Butler, PA), and the temperature was controlled at 37°C. For live-cell imaging, each cell represents a separate experiment.

### TIRF, Two-wavelength Imaging, and Parallax Microscopies

Live-cell imaging was conducted on one of two microscopes using total internal reflection fluorescence (TIRF) microscopy. For single wavelength imaging, a Leica DM IRB microscope was used with a Nikon 60x, Plan-apochromat 1.45 NA oil immersion objective and a Hamamatsu C4742-95 camera. A Melles Griot (Albuquerque, NM) 43 Series Argon Ion Laser was used to excite the fluorophore at 488 or 514 nm. Imaging of two-wavelengths and 3-dimensional spatial imaging using Parallax microscopy [36] was conducted on an inverted Nikon TE-2000U microscope with a Nikon 100x, Plan-achromat 1.49 NA oil immersion objective and a Photometrics Cascade-512B camera. Solid state 488-nm (Sapphire 488 LP, Coherent, Santa Clara, CA) and 561-nm (CrystaLaser, Reno, NV) lasers were used to excite eGFP and mCherry, respectively. Spectral separation of the emission was accomplished using a Dual-View (Photometrics, Tucson, AZ) system with an insert containing 565 dichroic, 580 LP, and 515/30 BP filters from Chroma (Bellows Falls, VT).

### Image Collection and Analysis

#### HA-GLUT4-eGFP and IRAP time courses (Figures 1, 2, S1, and S5)

Differentiated cells transfected with HA-GLUT4-eGFP or IRAP-pHluorin were stimulated with starvation medium alone or starvation medium +100 nM insulin. TIRF images were acquired at 1 frame per 10 s. Using a custom script written in MATLAB, intensity within the initial cell footprint was measured at each time point, and measurements were background-subtracted. For evaluation of transient intensity changes, measurements were binned in 0.5 minute intervals and intensity changes were scaled by dividing by the average intensity prior to insulin stimulation. The graph of intensity was normalized from 0, the average intensity prior to insulin addition, to 1, the average of the last minute of the plot. Cells scored as responding to insulin (fluorescence intensity increased by at least 10%) were averaged together, and the 95% confidence intervals were calculated. Approximately 78% of the cells morphologically distinguished as adipocytes responded to insulin (76 out of 97 cells), as assessed by changes in HA-GLUT4-eGFP or IRAP-pHluorin fluorescence.

Statistical analysis of the time courses was complicated by the lack of model for fitting our data. p-Values calculated at individual time points may not be statistically significant (two-tailed t-test; p>0.05) due to the heterogeneity in the magnitude of the insulin response. However, since there is a temporal relationship between time points, a new p-value, P, can be calculated as follows. For time points t_1_, t_2_, t_3_, …, t_n_, q = p_1_*p_2_*p_3_*…*p_n_, where p is the p-value at time point t and q is related to the new p-value, P = q(1−ln(q)+(ln(q))^2/2^! - … (ln(q))^n−1^/(n−1)!. This value, P, can also be calculated from the sum of the individual χ^2^ values at each time point: χ^2^ =  χ_1_^2^+ χ_2_^2^+ χ_3_^2^+ … + χ_n_^2^, with 2 n degrees of freedom.

#### Microtubule curvature and density time course

Differentiated cells transfected with GFP-tubulin, mCherry-tubulin, or 3xGFP-EMTB were stimulated by the addition of 100 nM insulin. TIRF images were acquired at 1 frame per 10 s. A subset of cells was co-transfected with HA-GLUT4-eGFP and mCherry-tubulin for temporal correlation analysis, and the dual-view insert was used to spectrally separate the fluorescence emissions. For quantification of microtubule density (Figure 2A), we performed image segmentation of the microtubules and then normalized to cell area. First, images were background-subtracted in ImageJ (version 1.44c), and the intensity was thresholded to create a binary image of the microtubules. Density was measured by dividing the microtubule area determined from the binary image by the total cell area. For each trace, measurements were binned in 0.5 min intervals. Density was normalized as described for HA-GLUT4-eGFP intensity, except that the average of the last 8 minutes was used for the final density value. Cells responding to insulin with a visible microtubule density increase were averaged together, and the 95% confidence interval was calculated.

For visualization and quantification of microtubule curvature (Figures 3A and S3), images were background-subtracted, and microtubule contours were outlined by hand using a spline fit in ImageJ. The cosine correlation function was calculated for each contour at least 3 µm in length using a custom script written in MATLAB. See supplement for details (Materials S1: Cosine correlation function, Figure S3). For Figure 3D, the CCF for microtubules at the indicated times post-insulin were averaged together. P- values were calculated by comparing the average CCF for the indicated times post-insulin at a defined contour length using a two-tailed t-test.

#### Parallax and 3-dimensional imaging of microtubules

Differentiated cells were transfected with 3xGFP-EMTB. TIRF images were acquired at 1 frame per 10 s using Parallax microscopy [36]. Microtubule contours for each pair of images were obtained using NeuronJ [61] (Figure 4). Contours were divided into discrete segments, and the z-coordinate was calculated for each segment using a custom script written in MATLAB. See supplement for details (Materials S1: Parallax time-lapse).

#### Correlation of fusion locations with microtubules

Differentiated cells were co-transfected with IRAP-pHluorin and either mCherry-tubulin for quantification or 3×GFP-EMTB for visualization and stimulated with 100 nM insulin. TIRF images were acquired several minutes following insulin addition at 20 frames per 1 s, and the dual-view insert was used to spectrally separate the fluorescence emissions. IRAP-pHluorin fluorescence was scored as a fusion event if the fluorescence appeared during acquisition of the video sequence and dispersed within the plane of the membrane during subsequent frames. Microtubule intensity was thresholded as described above and skeletonized in ImageJ to create a 1-pixel line locating the microtubules. Fusion location was determined, and the distance of the fusion to the nearest microtubule location was calculated using MATLAB. Due to photobleaching, analysis was limited to the first ∼15 s of the acquisition. To compare the distribution of fusion sites to a random distribution, ‘mock’ fusion locations (i.e., random locations) were randomly chosen within the area of the cell using a custom script written in MATLAB. Distance of the random location to the nearest microtubule location was calculated (n = 100 repetitions). Microtubule density was calculated by dividing the number of positive pixels in the binary image by the cell area. Cells were grouped according to microtubule density, and histograms of the fractions of events occurring within a given distance from a microtubule were generated. Error bars for fusions represent the standard deviation resulting from bootstrapping (n = 100 repetitions) the data.

#### Vesicle movement prior to fusion

Differentiated cells transfected with mCherry-IRAP-pHluorin were stimulated with 100 nM insulin. TIRF images were acquired as described for the preceding section. Vesicle movements prior to fusion were scored as long-distance if the vesicle moved ∼1 µm or more. Otherwise, vesicle movements were scored as not long-distance. Sometimes vesicles appeared to change velocity and direction repeatedly and rapidly, but these movements remained following nocodazole treatment, were less than 1 µm, and were judged not to be long-distance movements. When a fusion event could be detected but nearby vesicle density was too high, or mCherry fluorescence intensity of the IRAP construct was too dim, vesicle movements were scored as unknown.

## Supporting Information

Figure S1 **Time courses of HA-GLUT4-eGFP intensity and microtubule density increase.** Adipocytes (A and C) transfected with HA-GLUT4-eGFP or (B) co-transfected with HA-GLUT4-GFP and mCherry-Tubulin were serum-starved prior to stimulation with 100 nM insulin at t = 0 min. Images were acquired using TIRF microscopy at 1 frame per 10 s. (A) Addition of 100 nM insulin (black arrow) results in the increase in HA-GLUT4-eGFP in the TIRF illumination zone. Addition of starvation medium 5–10 min before addition of insulin (blue arrow, stdev indicated) does not result in a fluorescence increase. Plotted is the mean ±95% confidence interval (n = 8 cells). (B) In cells co-transfected with mCherry-tubulin and HA-GLUT4-eGFP, the time course of the microtubule density increase in the TIRF illumination zone precedes the increase in the intensity of HA-GLUT4-eGFP. Intensity or density increases for each cell were normalized from 0 to 1 as in Figure 2A. Plotted is the mean ±95% confidence interval (n = 4 cells). (C) Time courses of HA-GLUT4-GFP intensity increase are similar regardless of final fold intensity. Intensity was normalized from 0 to 1. Plotted in black is the average intensity across all cells, as plotted in Figure 2A (n = 13 cells). Plotted in blue, red, and green are the time courses for individual cells with final fold intensity values of 1.4, 2.0, and 2.5, respectively.
(EPS)

Figure S2 **Microtubule curvature in 3T3-L1 adipocytes.** (A) Curved microtubules are seen when visualized by TIRF via immunofluorescence of endogenous tubulin (clone DM1A) in fixed cells, or by direct observation of adipocytes transfected with GFP-tubulin, mCherry-tubulin, or 3xGFP-EMTB. Scale bar is 10 µm. Inset is 5 µm×5 µm. (B) TIRF images of an adipocyte transfected with mCherry-tubulin and treated with 2 µM nocodazole (added at t = 0) show curved microtubules. Thus, forces from microtubule polymerization are not responsible for bending. TIRF images have been background-subtracted. Scale bar is 10 µm. See Video S9. (C) TIRF image of adipocyte co-transfected with mCherry-tubulin and GFP-CC1 p150Glued showing the presence of curved microtubules. Scale bar is 5 µm. (D) TIRF image of adipocytes transfected with GFP-tubulin and treated with 1 µM cytoD or 20 µM latB for a minimum of 20 minutes showing curved microtubules. The treatment was sufficient to disrupt the actin cytoskeleton. TIRF images have been background-subtracted. Scale bar is 5 µm.
(EPS)

Figure S3 **Microtubule contours and cosine correlation function.** (A) Contours for a straight (red) and curved (blue) microtubule were replotted to share the same origin and initial orientation. Segments along the contour of the microtubule (s_n_) make an angle (θ_Sn_) with respect to the horizontal axis. (B) Cosine correlation function for microtubule contours shown in (A). Cos(θ_Sn+x_−θ_Sn_) was calculated for each pair of segments separated by contour distance ×(µm), and values for all pairs were averaged. See supplement for details (Materials S1: Cosine correlation function).
(EPS)

Figure S4 **Microtubule curvature does not correlate with acetylation in 3T3-L1 adipocytes.** 3T3-L1 cells were removed from culture dishes by trypsin treatment and replated in growth medium onto glass coverslips, fixed, and immunostained with α-tubulin (for visualization of total tubulin) and α-acetylated-tubulin antibodies. (A, Top) Epifluorescence images of an adipocyte stained for total (magenta) and acetylated (green) tubulin. The overlay shows regions of colocalization (white). Scale bar is 15 µm. (A, Bottom) Background-subtracted image of overlay with regions of interest highlighted in white boxes and expanded to the right. Scale bar is 15 µm. Expanded regions have dimensions of 5 µm×5 µm. (B) Epifluorescence images of an adipocyte (highlighted by an asterisk) flanked by two incompletely differentiated, fibroblast-like cells. (Bottom Right) The ratio of the background-subtracted image of α-acetylated-tubulin to the background-subtracted image of α-tubulin was calculated, and values were divided by the minimum value. White indicates the highest ratio of α-acetylated-tubulin to α-tubulin. Scale bar is 20 µm.
(TIF)

Figure S5 **Insulin stimulation increases the intensity of IRAP-pHluorin in the TIRF illumination zone.** Adipocytes transfected with IRAP-pHluorin were serum-starved and pre-treated with 0 µM (blue, n = 8 cells) or 10 µM nocodazole (red, n = 12 cells) prior to stimulation with 100 nM insulin at t = 0 min. Images were acquired using TIRF microscopy at 1 frame per 10 s. (A) Time course of the fold intensity increase. Plotted is the mean ±95% confidence interval. There is a significant difference (two-tailed t-test; p-value <0.05) between the two time courses at the half-time for IRAP-pHluorin intensity increase (t = 7 min, 0 µM Noc). Due to heterogeneity in the magnitude of the insulin response, a two-tailed t-test performed at t = 28 min does not yield a significant p-value (p-value >0.05). However, since there is a temporal relationship between time points, a new p-value, P, can be calculated by taking this relationship into consideration (see Methods). Comparing the last several minutes of the plateaus gives a significant difference (P<0.05) between the 0 µM Noc and 10 µM Noc time courses. (B) Time course of the intensity increase normalized from 0 (average intensity prior to insulin addition) to 1 (intensity at last minute of time course). Plotted is the mean ±95% confidence interval. Half-times are plotted (0 µM Noc, *t*_1/2_ = 7 min, solid black line; 10 µM Noc, *t*_1/2_ = 8.5 min, dashed black line).
(EPS)

Video S1 **Insulin stimulation increases the intensity of HA-GLUT4-eGFP at the cell surface.** Adipocyte transfected with HA-GLUT4-eGFP was serum-starved prior to stimulation with 100 nM insulin at t = 0 min. TIRF images were acquired at 1 frame per 10 s. Scale bar is 15 µm.
(MOV)

Video S2 **Microtubule density increases upon insulin stimulation.** Adipocyte transfected with mCherry-tubulin was serum-starved prior to stimulation with 100 nM insulin at t = 0 min. TIRF images were acquired at 1 frame per 10 s. Scale bar is 10 µm.
(MOV)

Video S3 **Microtubule density increases upon insulin stimulation.** Adipocyte transfected with 3xGFP-EMTB was serum-starved prior to stimulation with 100 nM insulin at t = 0 min. TIRF images were acquired at 1 frame per 10 s. Scale bar is 20 µm. Elapsed time is indicated in min:sec.
(MOV)

Video S4 **Microtubule loops are formed in 3T3-L1 adipocytes.** Adipocyte was transfected with mCherry-tubulin. TIRF images were acquired at 1 frame per 10 s. Elapsed time is indicated in min:sec. Scale bar is 2 µm.
(MOV)

Video S5 **Microtubule sliding occurs relative to other microtubules in 3T3-L1 adipocytes.** Adipocyte transfected with 3×GFP-EMTB was serum-starved prior to stimulation with 100 nM insulin. TIRF images were acquired ∼5 min following insulin stimulation at an acquisition rate of 20 frames per 1 s. Background-subtraction was performed. Elapsed time is indicated in min:sec. Scale bar is 2 µm.
(MOV)

Video S6 **Microtubule gliding can be observed at the surface of 3T3-L1 adipocytes.** Adipocyte transfected with GFP-tubulin was starved prior to stimulation with 100 nM insulin. TIRF images were acquired ∼10 minutes following insulin stimulation at 1 frame per s. Elapsed time is indicated in min:sec. Background-subtraction and smoothing were performed. Scale bar is 5 µm.
(MOV)

Video S7 **2-Dimensional view of microtubule dynamics.** Adipocyte transfected with 3xGFP-EMTB was imaged using Parallax microscopy. One of a pair of 2-dimensional images is shown. TIRF images were acquired at 1 frame per 10 s. Background-subtraction was performed. Elapsed time is indicated in min:sec. Scale bar is 2 µm. Video corresponds to Figure 4.
(MOV)

Video S8 **3-Dimensional view of microtubule dynamics.** Microtubule from Figure 4 and Video S7 is plotted as a series of (x,y,z) coordinates. Relative z-depth is color-coded with 0 (deep red) being the closest approach of the microtubule to the coverslip. For ease of viewing microtubule movements, frames have been interpolated in time. Elapsed time is 3 min 20 s.
(MOV)

Video S9 **Microtubule curvature and displacement dynamics at the cell surface remain following initial treatment with a low dose of nocodazole.** Adipocyte transfected with mCherry-tubulin was treated with 2 µM nocodazole at t = 0 min. TIRF images were acquired at 1 frame per 20 s. Scale bar is 10 µm.
(MOV)

Video S10 **IRAP-pHluorin fusions with the plasma membrane occur in proximity to microtubules.** Adipocyte transfected with mCherry-IRAP-pHluorin and 3xGFP-EMTB was serum-starved prior to stimulation with 100 nM insulin. TIRF images were acquired ∼4 minutes following insulin stimulation at an acquisition rate of 9 frames per 1 s with 488 nm excitation and a 530df30 BP filter. Elapsed time is indicated in min:sec. Scale bar is 5 µm.
(MOV)

Materials S1 **Supplemental materials and methods.** Additional information regarding cloning of constructs, calculation of cosine correlation functions, and processing of parallax time courses has been included along with methods for immunofluorescence and attempts at disrupting microtubule dynamics.
(DOC)
