# Supplementary figures and images for: Sites of Glucose Transporter-4 Vesicle Fusion with the Plasma Membrane Correlate Spatially with Microtubules
Source: PLoS One. 2012 Aug 20;7(8):e43662. doi: 10.1371/journal.pone.0043662 (PMC3423385; doi:10.1371/journal.pone.0043662)

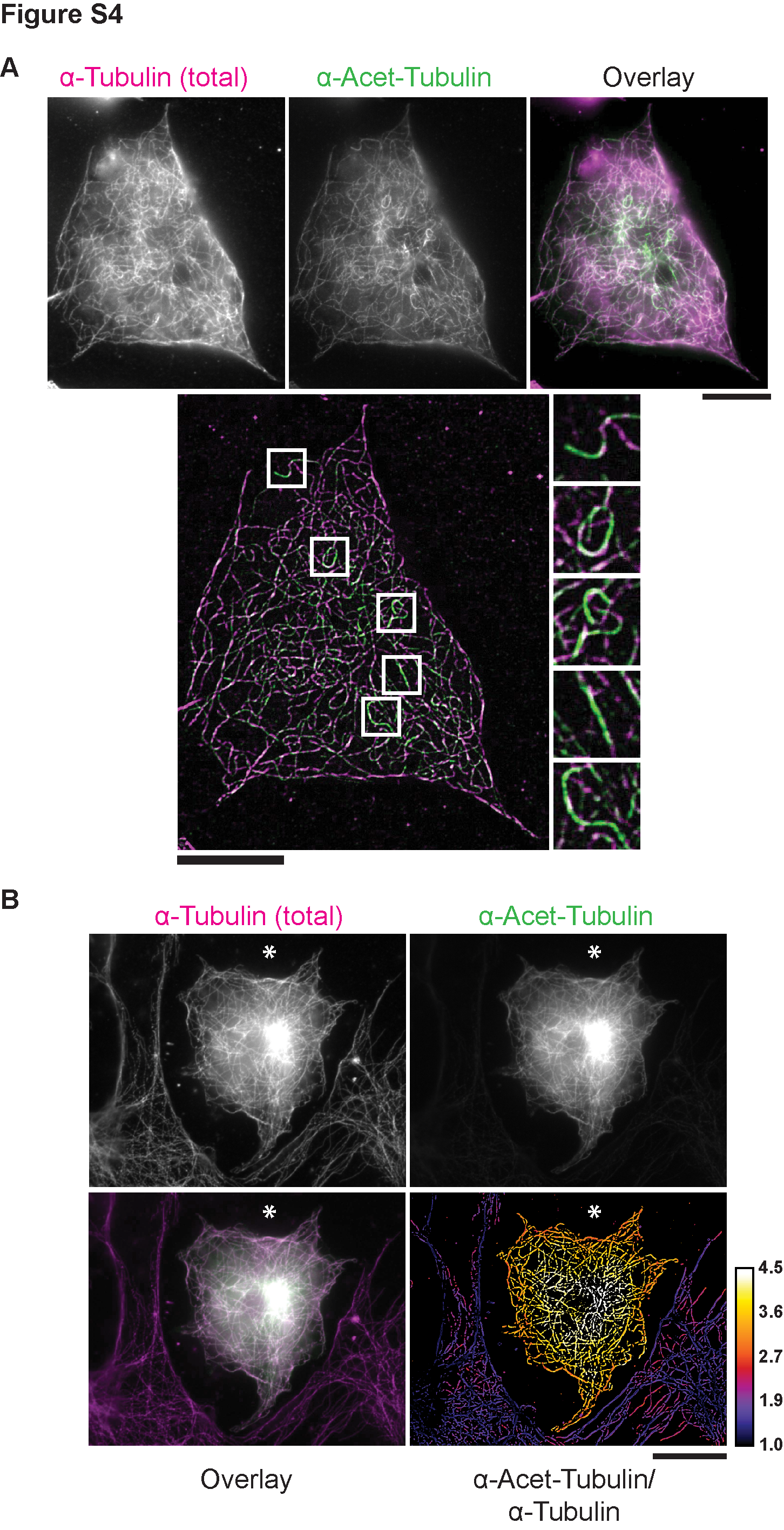

Supplement: Figure S4 — Microtubule curvature does not correlate with acetylation in 3T3-L1 adipocytes. 3T3-L1 cells were removed from culture dishes by trypsin treatment and replated in growth medium onto glass coverslips, fixed, and immunostained with α-tubulin (for visualization of total tubulin) and α-acetylated-tubulin antibodies. (A, Top) Epifluorescence images of an adipocyte stained for total (magenta) and acetylated (green) tubulin. The overlay shows regions of colocalization (white). Scale bar is 15 µm. (A, Bottom) Background-subtracted image of overlay with regions of interest highlighted in white boxes and expanded to the right. Scale bar is 15 µm. Expanded regions have dimensions of 5 µm×5 µm. (B) Epifluorescence images of an adipocyte (highlighted by an asterisk) flanked by two incompletely differentiated, fibroblast-like cells. (Bottom Right) The ratio of the background-subtracted image of α-acetylated-tubulin to the background-subtracted image of α-tubulin was calculated, and values were divided by the minimum value. White indicates the highest ratio of α-acetylated-tubulin to α-tubulin. Scale bar is 20 µm. (TIF) [file pone.0043662.s004.tif]
