# Supplementary material for: Sites of Glucose Transporter-4 Vesicle Fusion with the Plasma Membrane Correlate Spatially with Microtubules
Source: PLoS One. 2012 Aug 20;7(8):e43662. doi: 10.1371/journal.pone.0043662 (PMC3423385; doi:10.1371/journal.pone.0043662)
Supplement: Materials S1 — Supplemental materials and methods. Additional information regarding cloning of constructs, calculation of cosine correlation functions, and processing of parallax time courses has been included along with methods for immunofluorescence and attempts at disrupting microtubule dynamics. (DOC) [file pone.0043662.s016.doc]

# Materials S1. Supplemental Materials and Methods

**Cloning and Constructs**

For HA-GLUT4-mCherry (pJDM12), PCR primers were used to generate restriction sites flanking mCherry: 5’- GGGGTACCGCAATGGTGAGCAAGGGCGAGG -3’; 5’- GCACTCTAGATTACTTGTACAGCTCGTCCATGC -3’. For mCherry-IRAP-pHluorin (pJDM19), PCR primers were used to generate restriction sites flanking mCherry lacking a stop codon:
5’- GAAGCTAGCGACCATGGTGAGCAAGGGCGAGGAGG -3’; 5’- TGCAAGATCTTCTTGTACAGCTCGTCCATGCC -3’. Site-directed mutagenesis was then used to correct a base insertion in the reverse primer: 5’- GACGAGCTGTACAAGAGATCTCGAGCCACC -3’; 5’- GGTGGCTCGAGATCTCTTGTACAGCTCGTC -3’. Sequences were confirmed using the dideoxy method.

**Immunofluorescence**

The following primary antibodies were used for immunofluorescence of fixed cells: mouse monoclonal anti-tubulin clone DM1A, mouse monoclonal anti-acetylated tubulin antibody clone 6-11B-1, and pan-specific sheep anti-tubulin antibody (Cytoskeleton). Secondary antibodies were conjugated to Alexa 488 or Alexa 594 (Invitrogen/Molecular Probes).

For anti-α-tubulin staining (clone DM1A), cells were fixed in methanol + 1 mM EGTA pre-chilled to -20 °C for 10 minutes and permeabilized in PBS + 0.1% Igepal for 30 minutes at room temperature. For dual-staining of tubulin (pan-specific) and acetylated tubulin, cells were fixed in 4% paraformaldehyde in PBS for 15 minutes at room temperature and permeabilized in PBS + 0.1% Triton X-100 for 5 minutes at room temperature. Cells were blocked in PBS + 3% BSA. All antibodies were diluted in blocking buffer. Antibody incubations for anti-α-tubulin were 1 µg/mL for 1 hour at 37 °C for the primary and 1:500 for 1 hour at room temp for the Alexa 488-conjugated secondary. For dual-staining, primary antibodies were incubated sequentially
first with anti-tubulin (1:100) overnight at 4 °C followed by anti-acetylated-tubulin (1:100) for 30 minutes at 37 °C. Secondary antibodies were used at 1:300 and incubated for 30 minutes at 37 °C. Samples were mounted in ProLong Gold Antifade Reagent (Invitrogen) and imaged using TIRF (anti-α-tubulin) or epifluorescence (dual-staining) microscopies.

**Cosine correlation function**

The cosine correlation function was calculated using a custom script written in MATLAB. The microtubule was first divided into segments of length s (µm), and the angle theta (θ, rad) relative to the x-axis was determined for each segment. Any two segments are separated by a distance x (µm) along the contour of the microtubule. Their angles differ by a value Δθ = θ_Sn+x_ - θ_Sn_. Segments close to each other (i.e., x is small) will have similar values for theta, and the cosine of Δθ will be close to 1. However, when the contour distance between segments increases, the correlation between the two angles decreases.

When all values for cos(θ_Sn+x_ - θ_Sn_) for segments separated by a given distance x are averaged and plotted against x, the resulting cosine correlation function (CCF) decays with increasing x. How the CCF decays depends on the microtubule curvature. For a relatively straight microtubule (red), the CCF is nearly flat. For the curved microtubule (blue), <cos(θ_Sn+x_ - θ_Sn_)> decays relatively rapidly toward 0. Calculations of <cos(θ_Sn+x_ - θ_Sn_)> were limited to contour lengths of 0.45 times the filament length since the largest contour distances represent the average of just a few pairs of segments. See Figure S3.

**Parallax time-lapse**

The effective z calibration factor, which relates the separation in y of a pair of images to the relative distance in z, was obtained as described [1]. Briefly, Invitrogen 540/560 beads were tracked while an oscillation was applied to the stage in the z-dimension. The change in separation in y between pairs of bead images was plotted against the stage position in z. The slope of the line yields the apparent calibration factor, which is multiplied by a focal shift ratio to get the effective z calibration factor. The z-coordinate for each segment of the microtubule contour (see Methods) was calculated for each of the 21 frames of the acquisition. To aid in visualization of the final movie, microtubule contours were interpolated in time using a custom script written in MATLAB so that between each pair of frames calculated from acquired images are inserted two frames of interpolated data.

**Attempted disruption of microtubule dynamics**

Differentiated cells transfected with GFP-tubulin or mCherry-tubulin were treated with 2 µM nocodazole during imaging or pre-treated with 1 µM cytoD or 20 µM latB for a minimum of 20 minutes prior to imaging. Alternatively, differentiated cells were co-transfected with mCherry-tubulin and GFP-CC1 p150^Glued^. TIRF images were collected.

References

1. Sun Y, McKenna JD, Murray JM, Ostap EM, Goldman YE. (2009) Parallax: High accuracy three-dimensional single molecule tracking using split images. Nano Lett 9(7): 2676-2682.
